# Characterization and stabilization of GluLm and its application to deglycosylate dietary flavonoids and lignans

**DOI:** 10.1007/s00253-023-12956-9

**Published:** 2024-01-08

**Authors:** José Antonio Curiel, Ana Ruiz de la Bastida, Susana Langa, Ángela Peirotén, José María Landete

**Affiliations:** Food Technology Department, National Institute for Agricultural and Food Research and Technology (INIA-CSIC), Carretera de La Coruña Km 7.5, 28040 Madrid, Spain

**Keywords:** GH3 glucosidase, *Limosilactobacillus mucosae*, Isoflavones, Soy beverage, Lignans

## Abstract

**Abstract:**

This study describes the characterization of the recombinant GH3 aryl-β-glucosidase “GluLm” from *Limosilactobacillus mucosae* INIA P508, followed by its immobilization on an agarose support with the aim of developing an efficient application to increase the availability and concentration of flavonoid and lignan aglycones in a vegetal beverage. In previous studies, heterologous GluLm-producing strains demonstrated a great capacity to deglycosylate flavonoids. Nevertheless, the physicochemical properties and substrate spectrum of the enzyme remained unknown up to now. A high production of purified GluLm was achieved (14 mg L^−1^). GluLm exhibited optimal activity at broad ranges of pH (5.0–8.0) and temperature (25–60°C), as well as high affinity (*K*_*m*_ of 0.10 mmol L^−1^) and specific constant (86554.0 mmol L^−1^ s^−1^) against *p*-nitrophenyl-β-D-glucopyranoside. Similar to other GH3 β-glucosidases described in lactic acid bacteria, GluLm exhibited β-xylosidase, β-galactosidase, and β-fucosidase activities. However, this study has revealed for the first time that a GH3 β-glucosidase is capable to hydrolyze different families of glycosylated phenolics such as flavonoids and secoiridoids. Although it exhibited low thermal stability, immobilization of GluLm improved its thermostability and allowed the development of a beverage based on soybeans and flaxseed extract with high concentration of bioactive isoflavone (daidzein, genistein), lignan (secoisolariciresinol, pinoresinol, and matairesinol), and other flavonoid aglycones.

**Key points:**

*• Limosilactobacillus mucosae INIA P508 GluLm was purified and biochemically characterized*

*• Immobilized GluLm efficiently deglycosylated flavonoids and lignans from a vegetal beverage*

*• A viable application to produce vegetal beverages with a high content of aglycones is described*

**Supplementary Information:**

The online version contains supplementary material available at 10.1007/s00253-023-12956-9.

## Introduction

Dietary polyphenols have been described to induce health-promoting effects by exhibiting antioxidant, anti-inflammatory, anti-carcinogenic, anti-viral, anti-oxidation, anti-fatigue, anti-aging, and cardioprotective properties (Das et al. 2019; del Saz-Lara et al. 2022). Moreover, they reveal prebiotic activity and antimicrobial effects (Boubakeur et al. 2015). However, their functional properties and bioaccessibility are limited because they are mainly glycosylated, consequently exhibiting low bioavailability. However, their absorption can be enhanced through their transformation (deglycosylation) into more bioavailable aglycones (Peirotén et al. 2020a; Rasouli et al. 2017).

β-Glucosidases (EC 3.2.1.21) catalyze the hydrolysis of the alkyl- and aryl-β-glycosidic bonds of di- and oligosaccharides releasing glucose (Li et al. 2013). These enzymes are involved in other biological processes beyond the degradation of polysaccharides, such as defense against pathogens, cell wall metabolism, or cellular signaling (Bhatia et al. 2002). Given their applicability, the food industry uses β-glucosidases for several processes the most noteworthy being the synthesis of prebiotic oligosaccharides, the reduction of bitterness, and the improvement of organoleptic properties of vegetal foods and beverages through the deglycosylation of aromatic compounds (Acebrón et al. 2009; Del Pino-García et al. 2020; da Costa et al. 2018). Moreover, there is a growing trend towards the application of microbial β-glucosidases with the aim of improving the concentration and bioavailability of healthy polyphenol aglycones in plant-based foods (Otieno et al. 2005).

β-Glucosidase activity is widely distributed in lactic acid bacteria (LAB) and has also been described in yeast and fungi. From an industry point of view β-glucosidases are of great interest. Since LAB have been labeled with the qualified presumption of safety (QPS), several β-glucosidases from LAB have been explored for their biochemical properties (Del Pino-García et al. 2020). Regarding to this, some β-glucosidase-producer LAB strains have recently been reported for their ability to increase the concentration of bioactive aglycones from glycosylated isoflavones in fermented soy beverage (Ruiz de la Bastida et al. 2021; Ruiz de la Bastida et al. 2022). Among them, *Limosilactobacillus mucosae* INIA P508 stands out, from which the *glu*_913 putative gene was identified as a potential β-glucosidase and cloned in several LAB strains (Gaya et al. 2020). That study revealed that heterologous strains expressing the *glu*_913 gene from *L. mucosae* INIA P508 exhibited similar β-glucosidase activity to the gene-owner strain and were able to efficiently catalyze the deglycosylation of flavonoids (Gaya et al. 2020). However, the biochemical properties of the enzyme Glu_913 (henceforth GluLm) are still unknown. Therefore, because of (i) the great applicability of LAB β-glucosidases in the food industry and (ii) although GluLm has been genetically identified in *L. mucosae* its physicochemical properties and substrate specificity still remain unknown, this study describes the biochemical characterization and immobilization of the recombinant β-glucosidase GluLm from *L. mucosae* INIA P508 and its technological application on a vegetal beverage.

## Material and methods

### Bacterial strain and materials

*Limosilactobacillus mucosae* INIA P508 strain, isolated from breast-fed infant stool (Bravo et al. 2017), was propagated under strict anaerobic conditions (10% H_2_, 10% CO_2_ and 80% N_2_, Whitley DG250 Anaerobic Workstation, Don Whitley Scientific Ltd.) at 37°C in de Man, Rogosa, and Sharpe (MRS) broth. Strains of *Escherichia coli* DH5α and BL21 (DE3) were used for the propagation and expression of the pLATE31 expression vector (ALICator Ligation Scientific Cloning and Expression System, Thermo Fisher, USA), respectively. Both *E. coli* strains were cultured in Luria–Bertani (LB) broth under an agitation rate of 160 rpm at 37°C. The LB medium was supplemented with ampicillin and isopropyl-β-d-thiogalactopyranoside (IPTG) at a concentration of 100 μg mL^−1^ and 0.4 mmol L^−1^, respectively, when required.

### Architecture analysis, production, and purification of *L. mucosae* INIA P508 GluLm

The *L. mucosae* INIA P508 GluLm amino acid sequence (NCBI accession WP_143112948) was explored *in silico* to search for similar sequences, conserved domains, and phylogenetic analysis as described by Curiel et al. (2021) by using BLAST (NCBI), the InterPro database (EMBL), and Jalview 2.11.1.4 software, respectively. Furthermore, *gluLm* was PCR-amplified using the primers gluLmF (5′-**AGAAGGAGATATAACT*ATG****acaaaagtagacttgaattttgttgaggg*) and gluLmR (5′-**GTGGTGGTGATGGTGATGGCC***atctagttcattttaatttttgcagcaactc*) and subsequently cloned into pLATE31 following the ligation-independent cloning (LIC) procedure described by Curiel et al. (2022).

*E. coli* DH5α cells were transformed with LIC cloning product. The recombinant pLATE31 plasmids were isolated, and those, whose sequence confirmed the correct insertion of *gluLm*, were finally propagated to *E. coli* BL21 (DE3).

Recombinant *E. coli* BL21-pLATE31-*gluLm* cells were grown in 1 L of LB broth supplemented with ampicillin. Once the optical density reached 0.6 at 600 nm, and with the aim of inducing gluLm expression, IPTG was added to the culture, and the temperature was set to 22°C under agitation.

After 18 h, the cells were collected and washed twice. Subsequently, they were lysed by using the CelLytic™ B Plus Kit (Sigma-Aldrich, Germany) following the manufacturer’s instructions. Finally, the protein extract obtained, containing hyperproduced GluLm, was subjected to a Cytiva His GraviTrap affinity column (Cytiva, UK) as described by Del Pino-García et al. (2020). Because the protein eluted at increasing concentrations of imidazole, GluLm was dialyzed by using a 3500 cutoff membrane against 100 mmol L^−1^ sodium phosphate buffer, pH 7.0 at 4°C for 3 h. The grade of the enzyme purification was elucidated by 7.5% SDS-PAGE (Bio-Rad). The GluLm concentration was determined according to the Bradford method using a Quick Start Bradford protein assay (Bio-Rad) and bovine serum albumin as standard.

### GluLm β-glucosidase activity assay

The β-glucosidase activity of GluLm was quantified using *p*-nitrophenyl β-D-glucopyranoside (pNPG) as the substrate. The standard reaction (500 μL) was defined as the incubation of 5 μg of GluLm with 1 mmol L^−1^ pNPG in a 100 mmol L^−1^ sodium phosphate buffer, pH 6.5, at 37°C for 5 min. Subsequently, 500 μL of 0.5 mol L^−1^ Na_2_CO_3_ was added to stop the reaction. The absorbance exhibited was monitored using a Multiskan Spectrum microplate reader (Thermo Fisher, USA) at 400 nm. Previously, different concentrations of *p*-nitrophenol (pNP) ranging from 0.125 to 1 mmol L^−1^ were measured in order to prepare a standard curve. One unit (U) of GluLm activity was defined as the necessary amount of GluLm capable of catalyzing the release of 1 μmol pNP per minute under the conditions described. Reactions were carried out in triplicate for each analysis, and the results are shown as means ± standard deviations.

### Biochemical properties of GluLm

The effects of pH and temperature on the pNPG hydrolytic activity of GluLm were explored. The optimal pH of GluLm was determined with different pH buffers ranging in values from 3 to 10 at 37°C. The buffers (100 mmol L^−1^) used were acetic acid–sodium acetate (pH 3, 4, and 5), sodium phosphate (pH 6 and 7), Tris-HCl (pH 8 and 9), and sodium carbonate–bicarbonate (pH 10). The optimal temperature was assayed by incubating the recombinant GluLm in a 100 mmol L^−1^ pH 7.0 sodium phosphate buffer at the following temperatures: 25, 30, 37, 40, 50, and 60°C. Finally, the thermal stability was explored by incubating GluLm in a 100 mmol L^−1^ pH 7.0 sodium phosphate buffer at the following temperatures, 25, 30, 40, 50, and 60°C, at various times, 5, 15, and 30 min and 1, 2, 4, 8, 20, and 24 h. After incubations, the residual activity was measured following the standard reaction.

### Effect of chemicals on GluLm activity

The effect of different reagents on the GluLm activity was determined by the addition of diverse substances at a 1 mmol L^−1^ final concentration. The chemicals used were differentiated divalent and trivalent cations (CaCl_2_, CoCl_2_, HgCl_2_, MgCl_2_, MnCl_2_, ZnCl_2_, and FeCl_3_), detergent (Tween 80), and reducing (β-mercaptoethanol) and chaotropic (DMSO) agents. Furthermore, the repressive effect of glucose against GluLm was evaluated by adding different concentrations (0.1–1.0 mol L^−1^) to the reaction. Control was carried out following the standard reaction. The activity of GluLm was calculated relatively to the control sample without the reagent.

### Substrate specificity and kinetic properties of GluLm

The substrate specificity of GluLm was explored by using a 1 mmol L^−1^ final concentration of the following *p-*nitrophenyl derivatives *p*-nitrophenyl β-D-glucopyranoside (pNPG), *p*-nitrophenyl α-D-glucopyranoside, *p*-nitrophenyl β-D-galactopyranoside (pNPGAL), *p*-nitrophenyl α-D-galactopyranoside, *p*-nitrophenyl β-D-fucopyranoside (pNPBFU), *p*-nitrophenyl α-L-fucopyranoside (pNPAFU), *p*-nitrophenyl β-D-xylopyranoside (pNPXYL), and *p*-nitrophenyl α-D-xylopyranoside. The substrate specificity reactions were performed in the presence of 1 mmol L^−1^ CaCl_2_ for 1 h at 40°C and measured as described above.

In addition, the range of substrates of the recombinant GluLm was deepened using natural glucosylated substrates such as cellobiose, daidzin, genistin, oleuropein, quercetin-3-glucoside, quercetin-3-rutinoside, lactose, and trehalose. The glucose released by the β-glucosidase activity of GluLm from these substrates was determined using the Glucose Assay Kit (Megazyme) according to Del Pino-García et al. (2020). Furthermore, GluLm was also assayed using fucosylated oligosaccharides 2′-fucosyllactose and 3-fucosyllactose as natural substrates. The released fucose was monitored using the K-Fucose kit (Megazyme) as described by Curiel et al. (2022). The results were performed in triplicate and are shown as means ± standard deviations.

The enzyme kinetics of GluLm were studied using pNPG, pNPGAL, pNPBFU, pNPAFU, or pNPXYL as substrates at the concentrations range of 0.1 to 10 mmol L^−1^. The kinetic values of *K*_*m*_ and *V*_*max*_ were calculated by nonlinear regression analysis fitting to the Michaelis–Menten curves of the formation rates of pNP, and subsequently, *K*_*cat*_ and *K*_*cat*_ /*K*_*m*_ were obtained.

### GluLm stabilization and application in vegetal beverages

GluLm was stabilized through its immobilization to aldehyde group-activated agarose using the Aminolink Immobilization Kit (Thermo Scientific, USA). The multipoint and covalent binding of GluLm was carried out by the reaction between the primary amines of the exposed amino acids and the aldehyde groups exhibited by the activated agarose, following the manufacturer’s instructions. The immobilization yield and recovered activity were determined. Subsequently, the enzymatic stability was evaluated by incubating 11 μg of the immobilized GluLm derivative, equivalent to 5 μg of GluLm, in a 100 mmol L^−1^ sodium phosphate buffer, pH 7.0 at 50°C up to 24 h. The residual activity was measured following the standard reaction described above.

With the aim of elucidating the activity of stabilized GluLm on food matrices at the laboratory scale, it was incubated in the commercial soy beverage VegeDia (DIA Retail, Spain) enriched with lignans through 1% of flaxseed extract LinumLife EXTRA (Frutarom Netherlands BV, Netherlands) in order to explore the ability of GluLm to glycosylate isoflavones and lignans. The vegetal beverage was incubated in the presence of 1.1 mg of the immobilized GluLm derivative per liter of beverage at 40°C, 160 rpm for 4 h. After incubation, immobilized GluLm was removed from the beverage through a 0.45 μm filtration (Sartorius, Spain), and flavonoids and lignans were extracted following the procedure described by Peirotén et al. (2020b). Briefly, 1.5 ml of the sample was mixed with 500 μL of acetonitrile and vigorously shaken for 1 h and centrifuged at 13,500 rpm for 10 min. The filtered supernatants were analyzed by HPLC-DAD and HPLC-ESI/MS as described by Gaya et al. (2016). Quantification was carried out by means of external standard calibration curves.

## Results

### Amino acid sequence analysis of GluLm from *L. mucosae* INIA P508

**Fig. 1:**
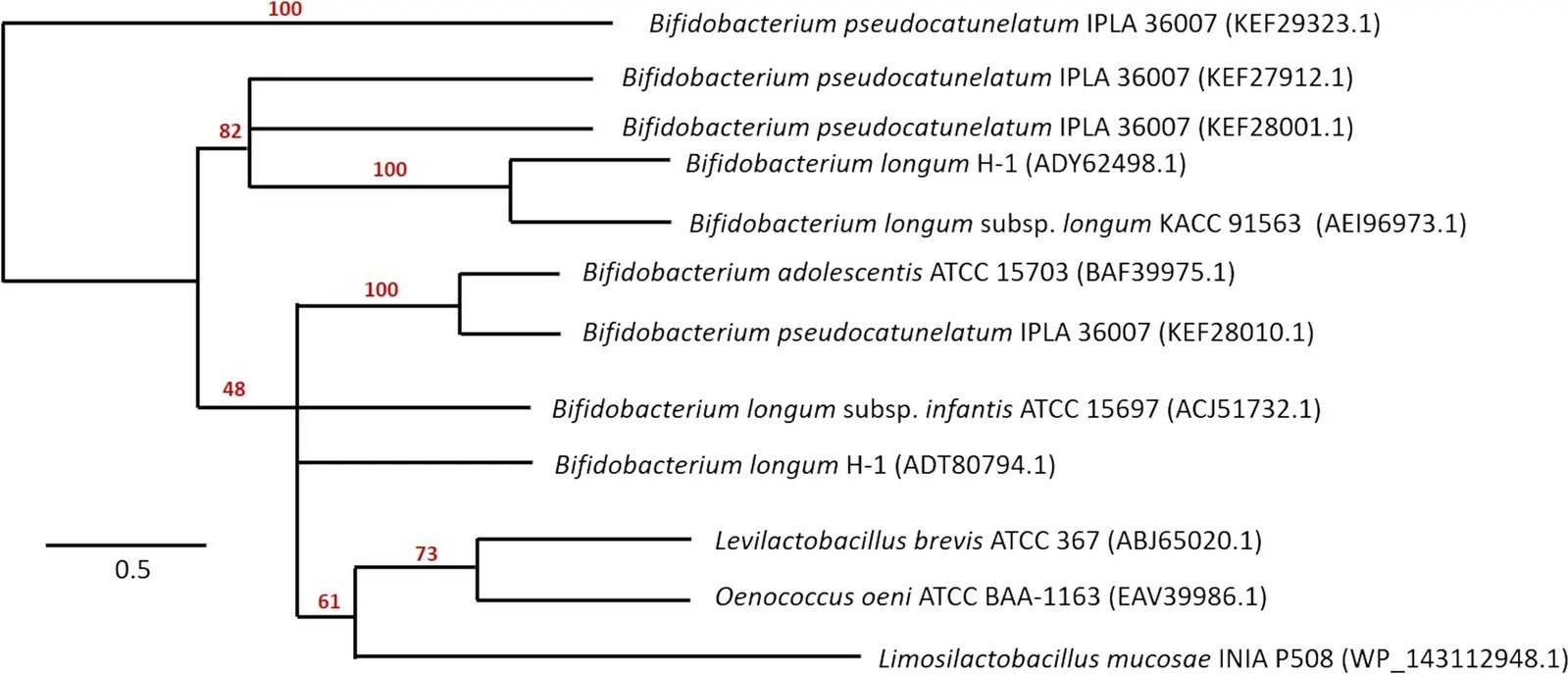
Phylogenetic analysis of different GH3 β-glucosidases characterized from *Bifidobacterium longum* subsp. *infantis* ATCC 15697 (ACJ51732.1; Matsumoto et al. 2015), *Bifidobacterium adolescentis* ATCC 15703 (BAF39975.1; Florindo et al. 2018), *Bifidobacterium longum* H-1 (ADY62498.1; ADT80794.1; Lee et al. 2011), *Bifidobacterium longum* subsp. *longum* KACC 91563 (ADY62498.1; Yan et al. 2018), *Bifidobacterium pseudocatenulatum* IPLA 36007 (KEF29323.1; KEF27912.1; KEF28001.1; KEF28010.1; Guadamuro et al. 2017), *Levilactobacillus brevis* ATCC 367 (ABJ65020.1; Michlmayr et al. 2009), *Oenococcus oeni* ATCC BAA-1163 (EAV39986.1; Michlmayr et al. 2010), and *Limosilactobacillus mucosae* INIA P508 (WP_143112948.1; this study). Phylogenetic tree is released from Jalview 2.11.1.4 software using the neighbor-joining method

Based on the conserved domain architecture explored using InterPro database (EMBL_EBI), GluLm should be clustered in the glycoside hydrolase Family 3 (GH3). In this context, and taking into account the sequence deposited in NCBI (WP_143112948), a search for similar sequences using BLAST (NCBI) highlighted a greater presence of putative β-glucosidases, revealing high identity to GluLm in different species of the same genus *Limosilactobacillus* as well as in *Oenococcus oeni* strains. However, these latter species showed less than 60% identity against the GluLm sequence (Table S1). Despite the high number of putative GH3 β-glucosidases, very few have been characterized from LAB. In this regard, the phylogenetical analysis was carried out using sequences of characterized GH3 β-glucosidases from LAB, *Levilactobacillus brevis* ATCC 367 (Michlmayr et al. 2009), *Oenococcus oeni* ATCC BAA-1163 (Michlmayr et al. 2010), and *Limosilactobacillus mucosae* INIA P508 (this study) and from bifidobacteria strains, *Bifidobacterium longum* subsp. *infantis* ATCC 15697 (Matsumoto et al. 2015), *Bifidobacterium adolescentis* ATCC 15703 (Florindo et al. 2018), *Bifidobacterium longum* H-1 (Lee et al. 2011), *Bifidobacterium longum* subsp. *longum* KACC 91563 (Yan et al. 2018), and *Bifidobacterium pseudocatenulatum* IPLA 36007 (Guadamuro et al. 2017; Fig. 1). The phylogram showed at least three distinguishable lineages of bifidobacterial GH3 β-glucosidases. While some sequences presented high interspecific homologies, such as the sequences *B. longum* ADY62498.1 and *B. longum* subsp. *longum* AEI96973.1, as well as *B. adolescentis* BAF39975.1 and *B. pseudocatenulatum* KEF28010.1, respectively, the sequence of *Bifidobacterium pseudocatenulatum* IPLA 36007 (KEF29323.1) was significantly different from the rest. On the other hand, the characterized β-glucosidases from LAB appear in a separate group from bifidobacterial, being GluLm significantly different and therefore, interesting for its biochemical elucidation (Fig. 1).

### Physicochemical characterization of GluLm

The gene *gluLm* was amplified from the *L. mucosae* INIA P508 strain using GluLmF and GluLmR primers, cloned into pLATE31 vector and hyperexpressed in *E. coli* BL21 (DE3) cells under the control of an IPTG inducible promoter. The sequence of *gluLm* present in the recombinant plasmid pLATE31-*gluLm* was analyzed, and its complete identity with the previously deposited (WP_143112948) was confirmed.

**Fig. 2:**
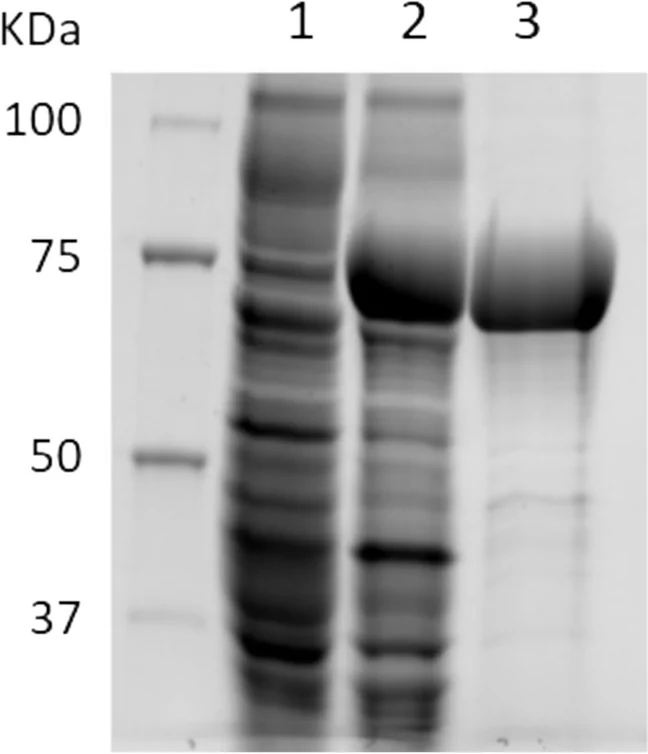
Analysis of the expression and purification of the GluLm enzyme from *L. mucosae* INIA P508. Cell extracts of the IPTG-induced *E. coli* BL21 (DE3) pLATE31, line 1; cell extracts of the IPTG-induced *E. coli* BL21 (DE3) GluLm, line 2; fraction of GluLm eluted after His-chelating affinity column, line 3. Unstained SDS-PAGE gel (7.5%) revealed through fluorescent detection (ChemiDoc MP Imaging System, Bio-Rad, Richmond, CA)

Hyperproduced GluLm was detected in the cell extracts from recombinant *E. coli* harboring the expression vector, unlike the control cells (Fig. 2). The 81-kDa molecular mass of recombinant GluLm corresponded to that inferred from the nucleotide sequence. Since *gluLm* was cloned together with an affinity poly-His tag, GluLm was purified by adding 200 mM imidazole to a His GraviTrap-chelating column (Fig. 2). Purified and dialyzed GluLm was subsequently tested for its β-glucosidase activity using pNPG as the substrate.

**Fig. 3:**
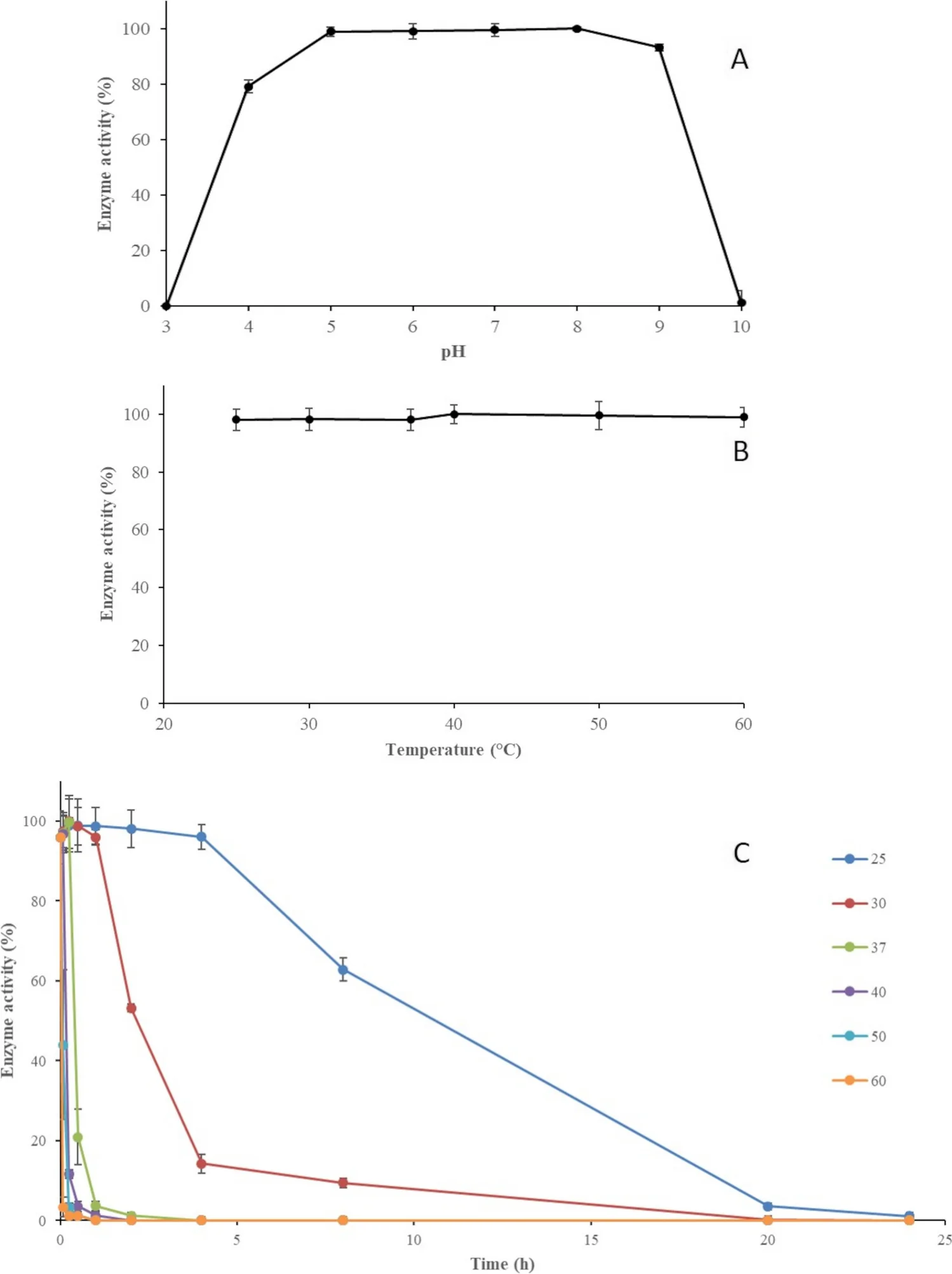
Biochemical properties of recombinant *L. mucosae* INIA P508 GluLm. **A** Relative activity of GluLm under different pH at 37°C for 5 min. **B** Relative activity of GluLm under different temperatures at pH 7.0 for 5 min. **C** Relative activity of GluLm at 37°C, at pH 7.0 for 5 min after different preincubation times (0–24 h) at 25°C (  ), 30°C (  ), 37 °C (  ), 40°C (  ), 50°C (  ), and 60°C (  ). The maximum activity was defined as the 100% in all cases

A total of 14 mg of recombinant GluLm with specific activity of 2.59 × 10^3^ U mg^−1^ was purified per 1 L of culture. Recombinant *L. mucosae* INIA P508 GluLm showed good performance in a wide pH range (5 to 8), also maintaining high activity at pH 4 and 9 (Fig. 3A). Recombinant GluLm also showed a wide range of optimal temperatures from 25 to 60°C (Fig. 3B). On the other hand, recombinant GluLm exhibited a high thermal sensibility, showing a pronounced decreased in activity after incubations of 4 h at 30°C or higher temperatures (Fig. 3C), and being unable to maintain any residual activity after 24 h at any of the temperatures tested (Fig. 3C).

The effects of some chemicals on GluLm were assayed (Table 1). The enzymatic activity of GluLm was slightly increased only by the presence of CaCl_2_ achieving the highest activity (106%). Among all the reagents assayed at final concentration of 1 mmol L^−1^ FeCl_3_, MgCl_2_ and MnCl_2_ did not affect the activity of GluLm. The remaining additives marginally reduced the GluLm activity to a range of 93.7 to 97.0%, with the exception of HgCl_2_ and the increasing concentrations of glucose, being HgCl_2_ and glucose 1 mol L^−1^ which reduced the GluLm activity below 10%.

**Table 1:** Analysis of the effects of different reagents on activity of recombinant *L. mucosae* INIA P508 GluLm

Chemical nature	Reagent	Relative activity (%)
Divalent cations	*Control	100.0 ± 1.5
	*CaCl_2_	106.1 ± 2.1
	*CoCl_2_	97.0 ± 2.6
	*HgCl_2_	9.9 ± 3.8
	*MgCl_2_	99.9 ± 0.1
	*MnCl_2_	99.5 ± 4.2
	*ZnCl_2_	95.8 ± 3.3
Trivalent cations	*FeCl_3_	99.0 ± 2.2
Detergent	*Tween 80	93.7 ± 2.1
Reducing agent	*β-Mercaptoethanol	94.0 ± 2.1
Chaotropic agent	*DMSO	94.5 ± 4.2
	Glucose 0.10 mol L^−1^	48.3 ± 5.7
	Glucose 0.25 mol L^−1^	21.8 ± 0.2
	Glucose 0.50 mol L^−1^	17.6 ± 1.3
	Glucose 1.00 mol L^−1^	7.8 ± 0.5

*Reagents at final concentration of 1 mmol L^−1^

As the Ca^2+^ ions increased the GluLm activity, a substrate specificity exploration was performed by using the optimal conditions observed for GluLm defined as follows: 100 mmol L^−1^ pH 7.0 phosphate buffer supplemented with 1 mmol L^−1^ CaCl_2_ at 40°C.

### Substrate specificity and kinetics of GluLm

The substrate specificity assay of *L. mucosae* INIA P508 GluLm was carried out by adding diverse *p*-nitrophenyl derivatives, as well as natural substrates such as cellobiose, daidzin, genistin, oleuropein, quercetin-3-glucoside, quercetin-3-rutinoside, trehalose, lactose, and fucosylated oligosaccharides, such as 2′-fucosyllactose and 3-fucosyllactose (Table 2).

**Table 2:** Substrate specificity analysis of recombinant *L. mucosae* INIA P508 GluLm.

Substrate (1 mmol L^−1^)	Relative activity (%)
*p*-Nitrophenyl β-D-glucopyranoside (pNPG)	100.0 ± 0.0^a^
*p*-Nitrophenyl α-D-glucopyranoside	2.2 ± 0.0^a^
*p*-Nitrophenyl β-D-galactopyranoside (pNPGAL)	68.8 ± 0.2^a^
*p*-Nitrophenyl α-D-galactopyranoside	0.3 ± 0.0^a^
*p*-Nitrophenyl β-D-fucopyranoside (pNPBFU)	95.2 ± 0.1^a^
*p*-Nitrophenyl α-L-fucopyranoside (pNPAFU)	26.6 ± 0.0^a^
*p*-Nitrophenyl β-D-xylopyranoside (pNPXYL)	96.8 ± 0.0^a^
*p*-Nitrophenyl α-D-xylopyranoside	0.1 ± 0.0^a^
Cellobiose	2.89 ± 0.41^b^
Daidzin	67.95 ± 1.58^b^
Genistin	64.13 ± 3.04^b^
Oleuropein	34.05 ± 3.73^b^
Quercetin-3-glucoside	11.13 ± 0.67^b^
Quercetin-3-rutinoside	1.40 ± 0.41^b^
Lactose	2.26 ± 1.30^b^
Trehalose	0.84 ± 0.74^b^
2’-Fucosyllactose	0.12 ± 0.09^c^
3-Fucosyllactose	0.00 ± 0.00^c^

^a^The indicated activity is relative to the concentration of *p*-nitrophenol released

^b^The indicated activity is relative to the concentration of glucose released

^c^The indicated activity is relative to the concentration of fucose released

According to the catalytic structure, GluLm could be included within the glycoside hydrolase family 3 (GH3) due to the presence of both the conserved GH3 N-terminal and C-terminal domains in its amino acid sequence (InterPro database EMBL_EBI; data not shown).

With the aim of biochemically determining that *gluLm* encodes a functional GH3 β-glucosidase, characterization analyses were performed using pNPG as the substrate (Table 2). Agreeing with what has been described for GH3 glucosidases, GluLm also showed a great activity when pNPXYL was used in the reaction (Table 2). In this context, interestingly, GluLm exhibited 68.8%, 95.2%, and 26.6% of relative activity when pNPGAL, pNPBFU, and pNPAFU were used as substrates, respectively (Table 2).

Despite GluLm activity was observed using pNPG, pNPGAL, and pNPAFU as substrates, the efficiency to hydrolyze cellobiose, quercetin-3-rutinoside, lactose, and 2′-fucosyllactose was quite low or inexistent (Table 2). However, moderate activity was exhibited against quercetin-3-glucoside (11.13%) and oleuropein (34.05%). On the other hand, GluLm greatly hydrolyzed daidzin (67.95%) and genistin 64.13%) into their corresponding aglycones (Table 2).

The kinetic properties of GluLm were also explored using pNPG, pNPGAL, pNPBFU, pNPAFU, and pNPXYL (Table 3). According to the substrate specificity results, GluLm revealed higher affinity to the substrate pNPG (*K*_*m*_ = 0.10 mmol L^−1^) than to any of the pNPXYL, pNPBFU, pNPGAL, or pNPAFU substrates (*K*_*m*_ = 0.76; 1.03; 2.81; and 2.82 mmol L^−1^, respectively). *V*_*max*_ was calculated in order to elucidate the specificity constants (*K*_*cat*_*/K*_*m*_) of GluLm against the assayed substrates, showing the highest specificity for the pNPG substrate (Table 3).

**Table 3:** Kinetics of GluLm from *L. mucosae* INIA P508

	*K*_*m*_ (mmol L^−1^)	*V*_*max*_ (μmol min^−1^)	*K*_*cat*_ (s^−1^)	*K*_*cat*_*/K*_*m*_ (mmol L^−1^ s^−1^)
pNPG	0.10 ±0.01	33.78 ±2.54	9120.0	86554.0
pNPGAL	2.81 ±0.18	0.86 ±0.05	117.0	41.9
pNPBFU	1.03 ±0.04	1.64 ±0.09	223.3	217.5
pNPAFU	2.82 ±0.24	0.06 ±0.00	7.8	2.7
pNPXYL	0.76 ±0.05	4.07 ±0.04	552.5	728.6

*pNPG*, *p*-nitrophenyl β-D-glucopyranoside; *pNPGAL*, *p*-nitrophenyl β-D-galactopyranoside; *pNPBFU*, *p*-nitrophenyl β-D-fucopyranoside; *pNPAFU*, *p*-nitrophenyl α-L-fucopyranoside; *pNPXYL*, *p*-nitrophenyl β-D-xylopyranoside

### Stabilization and hydrolytic activity of immobilized GluLm on a vegetal beverage

**Fig. 4:**
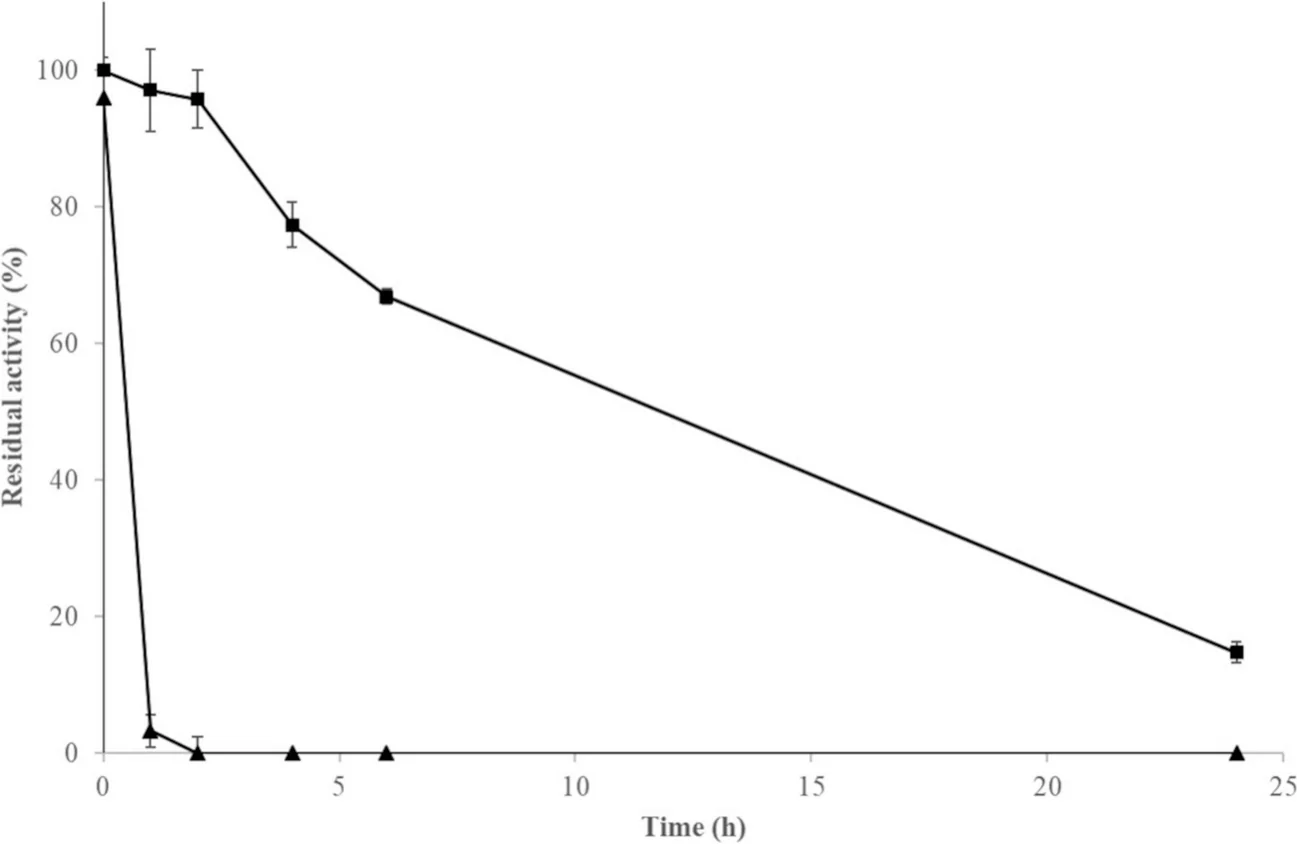
Thermostability of recombinant *L. mucosae* INIA P508 GluLm immobilized (■) and soluble state (▲) at 50°C. Values are represented by the relative residual activity.

GluLm was stabilized by immobilizing it through covalent and multipoint bonds with activated aldehyde groups on an agarose support. The GluLm derivative exhibited an immobilization yield of 99.82% and did not affect the activity as GluLm showed a recovered activity of 98.57% (data not shown). Furthermore, immobilized GluLm showed an improved enzymatic stability at 50°C compared to its soluble state, exhibiting more than 60% of residual activity after 6 h (Fig. 4).

**Fig. 5:**
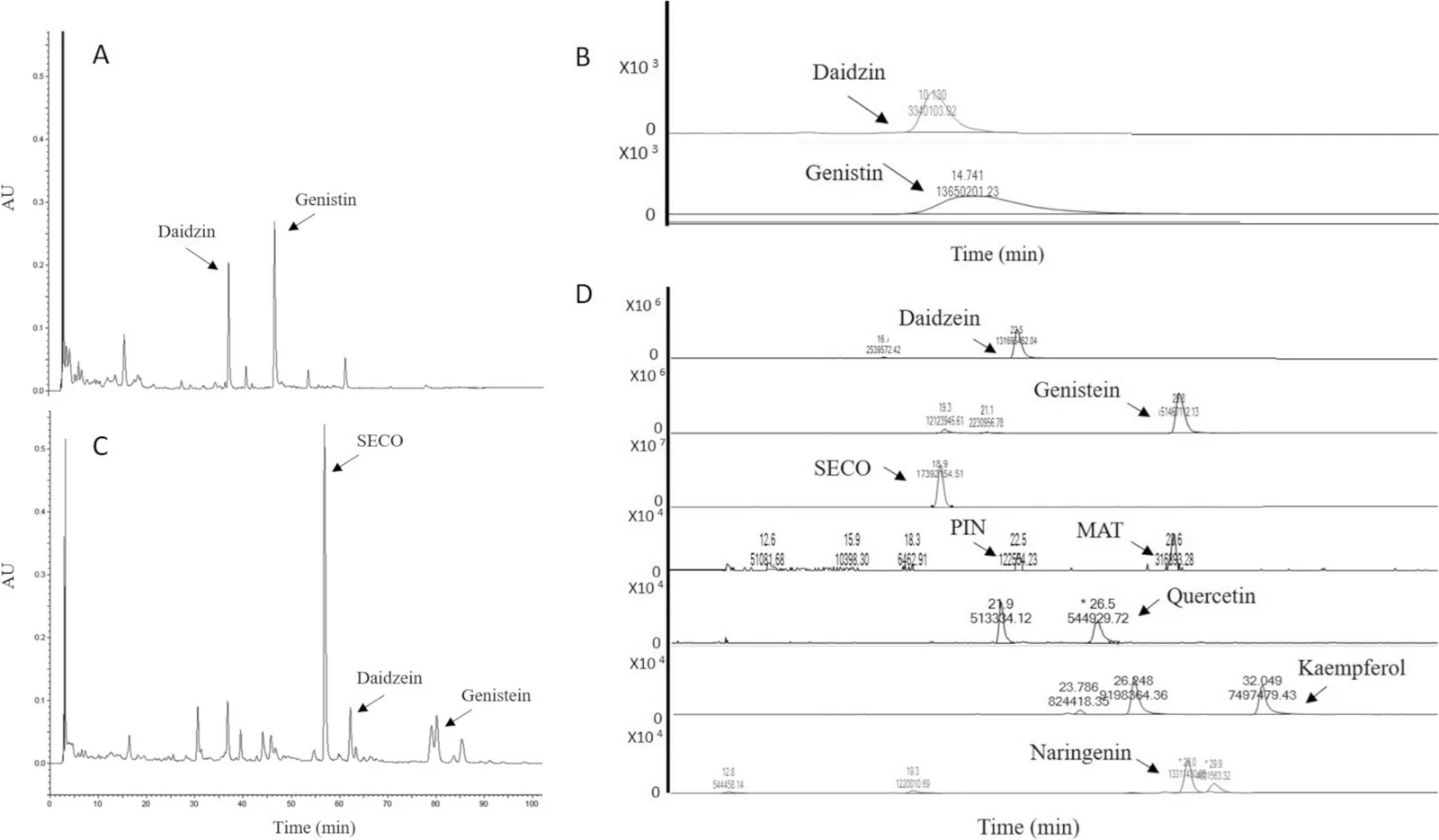
HPLC-DAD and HPLC-ESI/MS chromatograms of soy beverage supplemented with 1% of flaxseed extract (**A** and **B**, respectively) and after incubation with immobilized GluLm from *L. mucosae* INIA P508 (**C** and **D**, respectively)

With the aim of elucidating the capacity of stabilized GluLm to deglycosylate phenolic precursors in food matrices, it was incubated in a soy beverage enriched with 1% of flaxseed extract. The results from HPLC-DAD and HPLC-ESI/MS indicated the great efficiency of GluLm to completely transform the isoflavones daidzin and genistin into their corresponding aglycones daidzein and genistein, reaching concentrations of 312.99 ± 6.78 and 583.45 ± 1.91 μmol L^−1^ respectively (Table 4, Fig. 5C). Even though the addition of flaxseed extract to the soy beverage had no impact on the control observed by HPLC-DAD (Table 4, Fig. 5A), GluLm was able to transform the glycosylated lignans into their respective aglycones secoisolariciresinol (SECO), pinoresinol (PIN), and matairesinol (MAT), highlighting the great concentration of 3558.03 ± 15.87 μmol L^−1^ of SECO (Table 4, Fig. 5D). Finally, GluLm was able to deglycosylate other minor flavonoids present in the assayed beverage, observing increments of kaempferol and naringenin, as well as quercetin, which was not detected in the control beverage (Table 4, Fig. 5D).

**Table 4:** Polyphenols (μmol L^−1^) detected in soy beverage supplemented with flaxseed extract control and incubated with stabilized GluLm

Phenolics	Compounds	[M-H]- (m/z)	Control (μmol L^−1)^	GluLm (μmol L^−1)^
Isoflavones	Daidzin^*+^	415.1035	125.31 ± 1.82	n.d.
	Genistin^*+^	431.0894	379.03 ± 65.98	n.d.
	Daidzein^*+^	253.0506	n.d.	312.99 ± 6.78
	Genistein^*+^	269.0455	n.d.	583.45 ± 1.91
Lignans	SECO^*+^	361.2	n.d.	3558.03 ± 15.87
	MAT^*^	357.1	n.d.	0.13 ± 0.01
	PIN^*^	351.39	n.d.	0.05 ± 0.01
Flavonols	Quercetin^*^	301.04265	n.d.	0.23 ± 0.01
	Kaempferol^*^	285.0405	0.07 ± 0.01	14.89 ± 0.42
Flavanones	Naringenin^*^	271.256	0.09 ± 0.01	3.19 ± 0.03

*HPLC-ESI/MS and + HPLC-DAD analysis of phenolic compounds. *n.d.*, not detected

*SECO*, secoisolariciresinol; *PIN*, pinoresinol; *MAT*, matairesinol

## Discussion

In previous studies, we described the great ability of the *L. mucosae* INIA P508 strain to deglycosylate flavonoids (Peirotén et al. 2020a; Bravo et al. 2017; Rasouli et al. 2017). In order to identify the corresponding β-glucosidase enzyme responsible to catalyze the hydrolysis of glycosylated precursors, two genes *glu*_152 and *glu*_913 (*gluLm*), putatively identified as β-glucosidases from the genome of *L. mucosae* INIA P508, were cloned and recombinantly expressed in *Lactococcus lactis*, *Lacticaseibacillus casei*, *Streptococcus thermophilus*, and *Bifidobacterium breve* (Gaya et al. 2020). All the heterologous strains harboring *gluLm* were able to deglycosylate isoflavones and lignans supplemented in cow’s milk and from soy beverages supplemented with flaxseed extract (Gaya et al. 2020; Ruiz de la Bastida et al. 2021). Therefore, those results highlighted the confirmation of GluLm from *L. mucosae* INIA P508 as a β-glucosidase with potential interest in food technology. However, since the technological application of recombinant strains is limited, the aim of this study was to elucidate the biochemical properties and substrate specificity and improve the stability of GluLm on solid GRAS supports, with the aim of allowing the technological application of GluLm in food matrices.

With this objective, GluLm was recombinantly hyperproduced using the commercial pLATE31 expression vector and *E. coli* as the host and purified. The architecture analysis of GluLm revealed that it belongs to the GH3 family. However, unlike GH3 β-glucosidases mentioned in Fig. 1, GluLm exhibited a broad range of optimal temperatures and pHs (Florindo et al. 2018; Lee et al. 2011; Matsumoto et al. 2015; Michlmayr et al. 2009; Michlmayr et al. 2010; Yan et al. 2018), similarly to other glycosyl hydrolases explored, such as Pgb4 and Pgb6 from *L. plantarum* (Plaza-Vinuesa et al. 2022; Plaza-Vinuesa et al. 2023). Only in terms of pH, Blon0625 from *B. longum* subsp*. infantis* ATCC 15697 has been described to exhibit a wide range, similar to GluLm (Matsumoto et al. 2015) as well as GluD and GluE from *B. pseudocatenulatum* IPLA 36007 in terms of pH, and GluA regarding to the range of temperature (Guadamuro et al. 2017).

With regard to the thermal resistance, GluLm exhibited a low thermal stability after incubations of 4 h at 30°C or higher temperatures. Although there is no data regarding the thermostability of bacterial GH3 β-glucosidases, GluLm thermal resistance was lower than TtBgl3, a fungal GH3 β-glucosidase from *Trametes trogii* which preserved 90% of residual activity after 5 h of incubation at 50 °C (Qu et al. 2022). Although Ca^2+^ ions increased the β-glucosidase activity, GluLm was shown to be sensitive to the repressive action of increasing glucose concentrations like the BAL GH3 β-glucosidases characterized from *L. brevis* ATCC 367 and *O. oeni* ATCC BAA-1163 (Michlmayr et al. 2009; Michlmayr et al. 2010). Even though this effect was not exhibited by the β-glucosidase from *B. adolescentis* ATCC 15703 (Florindo et al. 2018), it was reported in several fungal GH3 glucosidases (Decker et al. 2001; Salgado et al. 2018).

GluLm kinetic values confirm a slightly better affinity to pNPG than the rest of GH3 β-glucosidases characterized from bifidobacteria and lactic acid bacteria. However, GH3 β-glucosidase from *B. longum* subsp. *infantis* ATCC 15697 presented a higher catalytic efficiency than GluLm against pNPXYL (Matsumoto et al. 2015). Concerning the substrate specificity, GluLm was assayed individually with different *p*-nitrophenyl derivatives and natural glucosylated phenolics. Given the unspecificity of GluLm to hydrolyze different synthetic substrates, unlike to the previous GH3 glucosidases described, pNPAFU was included in the characterization in order to elucidate the role of GluLm to hydrolyze prebiotics such as fuco-oligosaccharides. Hence, this is the first time where a GH3 β-glucosidase is reported to defucosylate substrates such as pNPAFU. This activity had previously been described in LABs and bifidobacteria, the latter being characterized by producing different α-L-fucosidase enzymes with different phylogenetic lineages (Curiel et al. 2021, and Curiel et al. 2022), but unlike GluLm, none of them had been clustered within the GH3 β-glucosidase family. Despite this, the phylogenetic study of bifidobacterial fucosidases showed a great diversity, and some of them could exhibit additional glycosidase activities other than fucosidase (Curiel et al. 2021), which could agree with the results obtained with GluLm. However, GluLm was unable to hydrolyze the most abundant fucosylated oligosaccharides in breast milk, such as 2′/3-fucosyllactose (Table 2). Nevertheless, this observation does not rule out the fucosidase activity of GluLm, verified with pNPAFU, but this needs further testing with other natural fucosylated substrates.

The results observed concerning to the chemical structure of the natural substrates assayed indicated that GluLm could be a GH3 aryl-β-glucosidase with a preference for 7-O glucoside bonds found in daidzin and genistin. However, GluLm also exhibited a moderate capacity to transform oleuropein into oleuropein aglycone. Oleuropein is a secoiridoid glucoside and the major bioactive compound found in olive leaves, which are consumed through commercial nutraceutical extracts (Nardi et al. 2017). Even though oleuropein has been described to exhibit healthy properties, its aglycone has greater protective effects against Alzheimer’s, breast cancer, inflammation, hyperglycemia, and oxidative stress (Xu et al. 2018).

Although recently it has been described the absence of β-glucosidase activity on *L. plantarum* (Plaza-Vinuesa et al. 2022, 2023), this strain is able to transform oleuropein into its aglycone (Landete et al. 2008; Michlmayr and Kneifel 2014), probably due to the presence of several intracellular phospho-β-glucosidases (Plaza-Vinuesa et al. 2022, 2023). However, since these enzymes require phosphorylated substrates, from an industrial point of view, the application of β-glucosidases such as GluLm would be much convenient to obtain the more bioavailable oleuropein aglycone, since it would avoid the cost of the prior phosphorylation of oleuropein.

Finally, in order to discriminate the cellular metabolism of recombinant strains GluLm producers (Gaya et al. 2020), purified GluLm was assayed by using natural flavonoids found in soy beverage. Although there is no evidence of LAB GH3 β-glucosidases being able to catalyze the transformation of isoflavones, GluLm efficiently hydrolyzed daidzin and genistin. Deglycosylation is a key step in the isoflavone metabolism, since their aglycones (daidzein and genistein) and subsequent metabolites transformed by gut microbiota (equol, 5-hydroxy-equol) are more bioavailable and bioactive than the glycosylated isoflavones present in food (Okabe et al. 2010; Mayo et al. 2019). Thus, there is a growing interest in strategies to enrich foods rich in isoflavones with isoflavone aglycones, such as soy products (Ruiz de la Bastida et al. 2021; Ruiz de la Bastida et al. 2022).

Since the greatest weakness found in GluLm was its thermal sensitivity, an effective stabilization process through enzymatic immobilization on a GRAS agarose support was carried out. Given that immobilization, in addition to improving the enzymatic thermostability (Fig. 4), allows the recombinant enzyme removal from the final food product through the filtration of the carrier support, immobilized GluLm was assayed on a soy beverage enriched with 1% of flaxseed extract with the aim of elucidating its effectiveness to deglycosylate flavonoids and lignans. The results indicated that the application of immobilized GluLm in the assayed beverage allowed the complete transformation of the glycosylated isoflavones into their respective bioavailable and physiologically active aglycones daidzin and genistin (Ruiz de la Bastida et al. 2023). The concentration imbalance that occurred between the transformed isoflavone glycosides and their respective aglycones produced has been observed in other studies and could be due to the possible existence of other precursors of these aglycones in the soy beverage (Chun et al. 2007; Ruiz de la Bastida et al. 2022; Xia et al. 2019), 10.1016/j.jff.2019.103549).

Taking into account the bioactive effects of lignans (Mikropoulou et al. 2019) and that they are also present in nature in their glycosylated forms, the activity of GluLm was tested on a soy beverage enriched with flax extract rich in lignans. Although the main lignan secoisolariciresinol diglucoside was not detected by any method described, a high concentration of SECO was observed. In addition, other aglycones were observed in lower concentration such as PIN and MAT. This observation coincides with the results obtained from similar beverages fermented by recombinant strains carrying GluLm (Gaya et al. 2020). However, the application of GluLm improved the concentration of the aglycones, mainly of SECO, which was produced in a concentration which was three times greater. Other minor flavonols and flavanones from the soybean beverage were found to be deglycosylated. Among them, quercetin was found agreeing with the substrate specificity assay performed in this work. Related to this, although the kaempferol and naringenin precursors were not tested, their deglycosylated forms appeared in the beverage, showing that GluLm is also able to produce these aglycones.

The results obtained from the application of immobilized GluLm in the beverage are similar to those described, although a lower concentration of isoflavone aglycones has been noted (Hati et al. 2020; Angelotti et al. 2020). Probably, this fact is due to the use of a beverage with a higher soy content (20%; Hati et al. 2020) or the application on a concentrated extract of isoflavones (Angelotti et al. 2020). Nevertheless, GluLm managed to transform all the daidzin and genistin content of the beverage. Moreover, this is the first study where the activity of a glucosidase against lignans in a food matrix has been confirmed.

In summary, this is the first study where GluLm from *L. mucosae* INIA P508, a GH3 aryl-β-glucosidase, is characterized by its ability to deglycosylate dietary flavonoids and lignans into their respective bioactive and bioavailable aglycones. Considering also its strong affinity to pNPG, GluLm should be considered as an unspecific GH3 β-glucosidase. Since the substrate specificity described here for GluLm has not been previously reported in LAB GH3 β-glucosidases, we propose its further exploration for the activities highlighted in this work against oleuropein and fucosylated substrates. Moreover, we also suggest the updating of GH3 family properties on the CAZy database, based on the results observed from GluLm.

Since thermal sensitivity of GluLm could hinder its industrial application, enzyme immobilization was able to ameliorate this weakness. In this regard, we would like to highlight that the immobilized GluLm activity remains stable after more than 12 months and it can be reused. Furthermore, this stabilization enabled the feasible technological application of the recombinant GluLm to produce a functional flavonoid and lignan aglycone-enriched beverage, considering that the GRAS carrier support of GluLm is easily removed through a simple filtering process.

## Supplementary information

ESM 1 (PDF 163 kb)

## Data Availability

The authors declare that the data supporting the findings of this study are available within the article and its supplementary information files.
