# Supplementary material for: Characterization and stabilization of GluLm and its application to deglycosylate dietary flavonoids and lignans
Source: Appl Microbiol Biotechnol. 2024 Jan 8;108(1):80. doi: 10.1007/s00253-023-12956-9 (PMC10774645; doi:10.1007/s00253-023-12956-9)
Supplement: Supplementary file 1 — (PDF 163 kb) [file 253_2023_12956_MOESM1_ESM.pdf]

Supplementary material

Journal:

Applied Microbiology and Biotechnology

Manuscript title:

Characterization, stabilization of GluLm and its application to deglycosylate dietary flavonoids and lignans

Names of the authors:

José Antonio Curiel, Departamento de Tecnología de Alimentos, Instituto Nacional de Investigación y Tecnología Agraria y Alimentaria (INIA-CSIC), Carretera de La Coruña Km 7.5, 28040, Madrid, Spain.

Ana Ruiz de la Bastida, Departamento de Tecnología de Alimentos, Instituto Nacional de Investigación y Tecnología Agraria y Alimentaria (INIA-CSIC), Carretera de La Coruña Km 7.5, 28040, Madrid, Spain.

Susana Langa, Departamento de Tecnología de Alimentos, Instituto Nacional de Investigación y Tecnología Agraria y Alimentaria (INIA-CSIC), Carretera de La Coruña Km 7.5, 28040, Madrid, Spain.

Ángela Peirotén, Departamento de Tecnología de Alimentos, Instituto Nacional de Investigación y Tecnología Agraria y Alimentaria (INIA-CSIC), Carretera de La Coruña Km 7.5, 28040, Madrid, Spain.

José María Landete, Departamento de Tecnología de Alimentos, Instituto Nacional de Investigación y Tecnología Agraria y Alimentaria (INIA-CSIC), Carretera de La Coruña Km 7.5, 28040, Madrid, Spain.

Author to whom correspondence should be addressed: joseantonio.curiel@inia.csic.es; Tel.:

+34 91 347 68 86

List of supplementary materials:

Table S1. Similar sequences of GluLm analyzed by BLAST

Organism	Cover	Percent Identity	Accesion length	Accession
Limosilactobacillus mucosae	100%	100.00%	735	WP_143112948.1
Limosilactobacillus mucosae	100%	98.78%	735	WP_048345478.1
Limosilactobacillus mucosae	100%	98.64%	735	MBN2900744.1
Limosilactobacillus mucosae	100%	98.10%	735	WP_006500330.1
Limosilactobacillus mucosae	100%	95.65%	735	WP_180305058.1
Limosilactobacillus mucosae	100%	95.51%	735	WP_109588344.1
Lactobacillus sp.	100%	95.51%	735	HAM87287.1
Limosilactobacillus mucosae	100%	95.37%	735	WP_128513137.1
Limosilactobacillus mucosae	100%	95.37%	735	WP_033934378.1
Lactobacillus sp. MRS-253-APC-2B	100%	95.37%	735	WP_169461210.1
Limosilactobacillus mucosae	100%	90.75%	733	WP_056968697.1
Limosilactobacillus mucosae	100%	90.07%	735	WP_074505191.1
Candidatus Limosilactobacillus intestinipullorum	97%	70.43%	735	HJA22365.1
Limosilactobacillus antri	99%	68.94%	735	WP_007123550.1
Limosilactobacillus fermentum	97%	68.76%	759	MBS6067744.1
Limosilactobacillus panis	99%	68.44%	735	WP_152666511.1
Limosilactobacillus fermentum	98%	68.09%	726	KRN09458.1
Limosilactobacillus fermentum	99%	68.03%	734	WP_057728150.1
Limosilactobacillus fermentum	99%	68.03%	734	WP_062813394.1
Limosilactobacillus reuteri	99%	67.98%	735	WP_153702678.1
Limosilactobacillus oris	99%	67.98%	735	AMS07885.1
Limosilactobacillus oris	99%	67.85%	735	WP_194176945.1
Limosilactobacillus oris	99%	67.85%	735	WP_056984569.1
Limosilactobacillus oris	99%	67.71%	735	WP_003715911.1
Limosilactobacillus panis	99%	67.62%	735	WP_222259238.1
Limosilactobacillus reuteri	99%	67.44%	735	WP_134907017.1
Limosilactobacillus balticus	99%	67.44%	735	WP_182589157.1
Limosilactobacillus balticus	99%	67.44%	735	WP_182585977.1
Limosilactobacillus oris	99%	67.44%	735	WP_191363958.1
Limosilactobacillus reuteri	99%	67.17%	735	WP_122481902.1
Limosilactobacillus pontis	99%	66.76%	738	WP_104687862.1
Lactobacillus sp. Marseille-P3519	99%	66.58%	733	WP_076460733.1
Candidatus Limosilactobacillus excrementigallinarum	99%	65.67%	734	HJA46881.1
Limosilactobacillus rudii	99%	65.31%	733	WP_182597149.1
Limosilactobacillus fermentum	99%	64.85%	734	WP_035423528.1
Limosilactobacillus fermentum 28-3-CHN	99%	64.85%	740	EEX26469.1
Lactobacillus rodentium	99%	64.44%	734	WP_117118272.1
Lactobacillus sp.	99%	64.31%	734	MBD5432054.1
Limosilactobacillus agrestis	99%	64.03%	734	WP_182577622.1
Limosilactobacillus agrestis	99%	63.90%	734	WP_182600480.1
Weissella soli	99%	63.72%	736	WP_070229931.1
Weissella soli	99%	63.59%	736	WP_147152687.1
Limosilactobacillus secaliphilus	99%	63.47%	732	WP_057741166.1
Lentilactobacillus kisonensis DSM 19906 = JCM 15041	98%	58.96%	733	KRL20094.1
Lentilactobacillus kisonensis	99%	58.38%	740	WP_056949663.1
Lentilactobacillus kisonensis	99%	58.24%	740	WP_008858032.1
Oenococcus sicerae	97%	58.14%	718	VDK14753.1
Weissella sp. X0278	99%	58.03%	738	WP_165314570.1

Weissella paramesenteroides	99%	58.03%	738	WP_150190005.1
Secundilactobacillus collinoides	97%	57.98%	740	WP_056996384.1
Weissella hellenica	99%	57.89%	738	WP_147161790.1
Paucilactobacillus kaifaensis	99%	57.78%	741	WP_137596652.1
Lentilactobacillus sp. IMAU80584	99%	57.55%	740	WP_217163579.1
Lactobacillus panisapium	99%	57.12%	744	WP_220234737.1
Secundilactobacillus silagincola	99%	56.91%	739	WP_098826414.1
Oenococcus oeni	97%	56.89%	724	WP_071440276.1
Oenococcus oeni	97%	56.75%	727	WP_071417174.1
Oenococcus oeni	97%	56.75%	725	WP_071438476.1
Oenococcus oeni	97%	56.75%	726	WP_071417799.1
Oenococcus oeni	97%	56.75%	726	WP_071450668.1
Oenococcus oeni	97%	56.75%	726	OIK86960.1
Lactobacillus sp. W8092	98%	56.34%	738	WP_198204570.1
Oenococcus oeni	98%	56.28%	728	WP_071426374.1
Oenococcus oeni	98%	56.21%	729	WP_071439117.1
Oenococcus oeni	99%	56.05%	731	WP_071428561.1
Oenococcus oeni	99%	55.98%	734	WP_071436882.1
Oenococcus oeni	99%	55.98%	733	WP_071425160.1
Oenococcus oeni	99%	55.98%	732	WP_071458314.1
Oenococcus oeni	99%	55.95%	737	WP_096866762.1
Oenococcus oeni	99%	55.95%	737	WP_032811489.1
Oenococcus oeni	99%	55.95%	736	WP_071426169.1
Oenococcus oeni	99%	55.95%	737	WP_071445896.1
Oenococcus oeni	99%	55.95%	737	WP_002816431.1
Oenococcus oeni	99%	55.95%	737	WP_032807792.1
Oenococcus oeni	99%	55.81%	737	WP_002822087.1
Oenococcus oeni	99%	55.81%	737	WP_032826036.1
Oenococcus oeni	99%	55.81%	737	AIZ50375.1
Oenococcus oeni	99%	55.81%	737	WP_002823925.1
Oenococcus oeni	99%	55.81%	737	WP_096861063.1
Oenococcus oeni	99%	55.81%	737	WP_002819385.1
Oenococcus oeni	99%	55.81%	737	WP_071451324.1
Oenococcus oeni	99%	55.81%	737	WP_032808890.1
Oenococcus oeni	99%	55.75%	737	WP_071436957.1
Oenococcus oeni	99%	55.68%	737	AIZ50377.1
Oenococcus oeni	99%	55.68%	737	WP_071420054.1
Oenococcus oeni	99%	55.68%	737	WP_186432042.1
Oenococcus oeni	99%	55.68%	737	AIZ50376.1
Oenococcus oeni	99%	55.68%	737	WP_071435874.1
Oenococcus oeni	99%	55.68%	737	WP_002826096.1
Oenococcus oeni	99%	55.68%	737	WP_096866540.1
Oenococcus oeni	99%	55.68%	737	OIK61749.1
Oenococcus oeni S22	99%	55.68%	737	KGH55536.1
Oenococcus oeni	99%	55.68%	737	WP_071419412.1
Oenococcus oeni	99%	55.68%	737	WP_032817801.1

Oenococcus oeni	99%	55.59%	740	WP_186422090.1
Oenococcus oeni	99%	55.54%	737	WP_002823497.1
Oenococcus oeni	99%	55.54%	737	WP_071437224.1
Oenococcus oeni	99%	55.41%	737	WP_032822300.1
Oenococcus oeni	99%	55.41%	737	WP_011677766.1
Oenococcus oeni	99%	55.41%	737	AIZ50378.1
